# Numerical–Experimental Analysis of Polyethylene Pipe Deformation at Different Load Values

**DOI:** 10.3390/ma14010160

**Published:** 2020-12-31

**Authors:** Adam Gnatowski, Agnieszka Kijo-Kleczkowska, Mateusz Chyra, Dariusz Kwiatkowski

**Affiliations:** 1 Department of Technology and Automation, Faculty of Mechanical Engineering and Computer Science, Czestochowa University of Technology, Armii Krajowej 21, 42-201 Czestochowa, Poland; mateuszchyra@wp.pl (M.C.); kwiatkowski@ipp.pcz.pl (D.K.); 2 Department of Thermal Machinery, Faculty of Mechanical Engineering and Computer Science, Czestochowa University of Technology, Armii Krajowej 21, 42-201 Czestochowa, Poland; kijo@imc.pcz.pl

**Keywords:** polyethylene pipe, mechanical properties of polyethylene, resistance strain, computer simulation

## Abstract

Polymer pipes are used in the construction of underground gas, water, and sewage networks. During exploitation, various external forces work on the pipeline, which cause its deformation. In the paper, numerical analysis and experimental investigations of polyethylene pipe deformation at different external load values (500, 1000, 1500, and 2000 N) were performed. The authors measured strains of the lower and upper surface of the pipe during its loading moment using resistance strain gauges, which were located on the pipe at equal intervals. The results obtained from computer simulation and experimental studies were comparable. An innovative element of the research presented in the article is recognition of the impact of the proposed values of the load of polyethylene pipe on the change in its deformation.

## 1. Introduction

Polyethylene plastics are used in various branches of global industry, mainly in extrusion and injection technology in the form of pipes, foils, and various types of packaging. The chemical, physical, mechanical and aesthetic properties of polymer materials depend on the conditions of use: temperature, load time, type of deformation, atmospheric conditions, UV radiation, design solutions, soil parameters in which the pipeline works, and external forces, e.g., car traffic [1,2,3,4,5,6,7,8,9,10].

Polymer pipes are used in the construction of underground gas, water and sewage networks. This is due to their low weight compared to, e.g., steel pipes, which makes their transport and assembly much easier. Furthermore, polymer pipes are characterized by chemical inertness and very good mechanical properties [11,12,13].

Polymer pipe is a flexible material; therefore, it can be deformed at different external loads. The pipeline exploited in the soil react to the loads by deformation of its surfaces and by change in its cross-section. The value of the pipe deformation depends on the value of vertical force acting on the pipe and on the type and degree of soil compaction in which the pipeline is used [14,15,16]. The pipeline should be properly backfilled in the soil, and it must be on an even, uniform surface, free of large and sharp stones. During exploitation, the pipe is subjected to various loads (for example, the weight of the ground, buildings, road and rail traffic, embankments, and other objects).

At high loads, failure can occur due to the pipe wall breakage. A particularly dangerous case, from the point of view of the mechanical strength of the pipe, is pipeline installation at construction sites. At this stage, there is no working pressure in the pipe, which can cause it to stiffen. Furthermore, excavators, earth-filled lorries, move on such sites. High loading of pipelines can be caused by excessive soil layers directly above the pipe. This may lead to significant pipe deformation and, consequently, to pipeline damage even before its exploitation [17,18,19].

For this reason, it is essential to conduct experimental studies to prevent pipeline failure in real-life conditions. In regard to polyethylene material use, its thermal parameters are also very important [20,21,22,23].

In the paper, numerical analysis and experimental investigations of polyethylene pipe deformation at various external load values were performed. The studies were carried out on a specially designed test stand. The lateral strains on the lower and upper surface of the pipe were measured at the following pipe loadings: 500, 1000, 1500, and 2000 N.

## 2. Materials and Methods

In this paper, numerical analysis and experimental investigations of pipe deformation under the influence of various external load values were performed.

### 2.1. Numerical Analysis of the Influence of External Load on the Deformation of Polyethylene Pipe

Computer simulation was carried out for a high-density polyethylene pipe (HD-PE) loaded by evenly distributed soil and an external force on the central part of the pipe surface. The numerical analysis was performed by using the ADINA System 9.3.4 (ADINA R & D, Inc., 71 Elton Avenue, Watertown, MA 02472, USA) program. The spatial model of the system includes a soil block with approximate dimensions of 400 mm × 3100 mm × 400 mm in which the analyzed pipe is located. Computer simulation was carried out for the pipe with the following approximate dimensions: outer diameter of 40 mm, wall thickness of 3.7 mm, and length of 2300 mm. The pipe was placed in soil at a depth of 315 mm. In the central part of the model’s upper surface, an evenly distributed load was placed over an area of 320,000 mm^2^. Four load cases, 500, 1000, 1500, and 2000 N, were considered. The following boundary conditions were adopted in the tests:

- Restraining of the system’s lower surface;
- Restraining of the system’s side surfaces;
- No restraint of the pipe.

**Figure 1:**
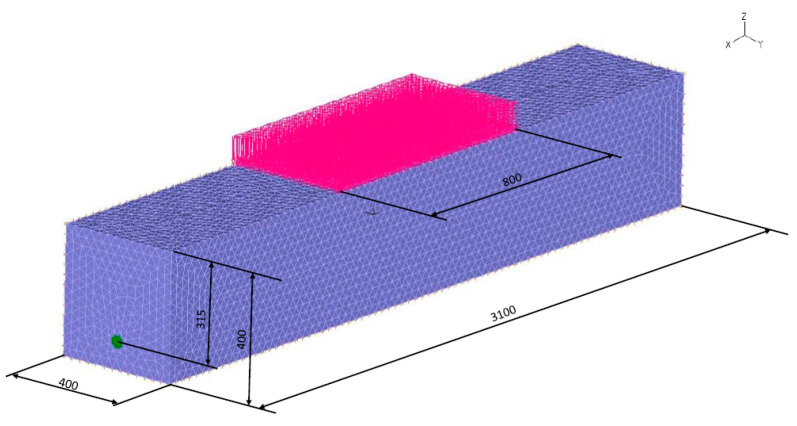
Diagram of the numerical model of the pipe–soil system. (Units in mm)

A diagram of the pipe–soil model is shown in Figure 1.

**Figure 2:**
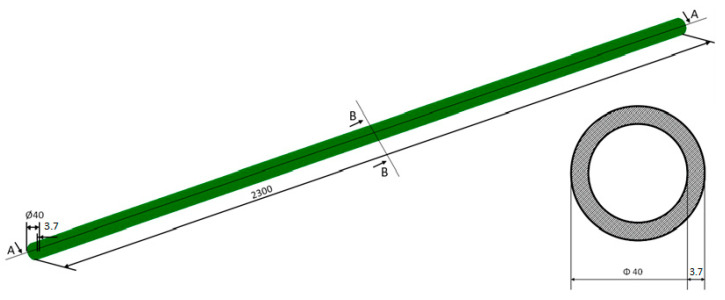
Model of the analyzed pipe. (Units in mm).

The dimensioned pipe model with the marked section plane is shown in Figure 2.

The tested model of the pipe–soil system consists of 237,766 finite elements (75,599 for the pipe; 162,167 for the soil) and 377,615 nodes. The soil was modeled with the use of the elastic–ideally plastic Coulomb–Mohr model, which is one of the most frequently used models in soil numerical descriptions [24,25,26,27]. The elastoplastic models describe the state of deformation and soil load in the zones subject to the limit state. In the Coulomb–Mohr model, the limit state is the same as the plastic surface. In the stress space, the yield surface for the Coulomb–Mohr model is defined by the following relationship [24]:

(1) $$\frac{1}{2}\left(\sigma_{1}-\sigma_{3}\right)+\frac{1}{2}\left(\sigma_{1}+\sigma_{3}\right)\sin\Theta -c\;\cos\Theta =0$$

where:

σ_1_, σ_2_, σ_3_—main stresses, MPa;
  θ—internal friction angle of soil, ^o^;
  c—cohesion, MPa.

**Figure 3:**
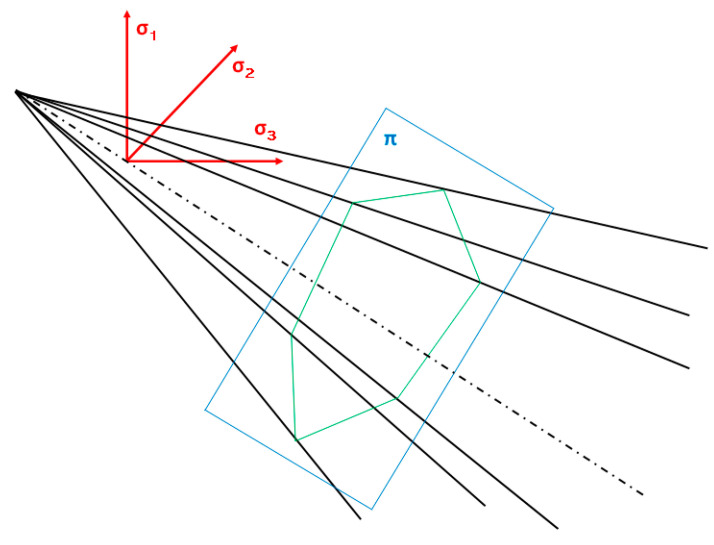
Plasticity area in the Coulomb–Mohr model.

In the spaces σ_1_, σ_2_, and σ_3_, the plastic area is limited by the side surfaces of the pyramid with the base of the hexagon (Figure 3), whose side lengths and angles between them change with the value of the internal friction angle.

The following geotechnical parameters were used in the computer simulation [28]:

- Compressibility modulus *p =* 20 MPa;
- Poisson’s ratio v = 0.32;
- Volumetric weight w = 0.000018 N/mm^3^;
- Cohesion c = 0.017 MPa;
- Internal friction angle θ = 17°.

The pipe was built on the basis of the elastic–isotropic material model. This model requires the definition of data such as Young’s modulus and Poisson’s ratio. In the discussed case, the following material parameters were adopted [29]:

- Young’s modulus E = 1000 MPa;
- Poisson’s ratio v = 0.46.

**Figure 4:**
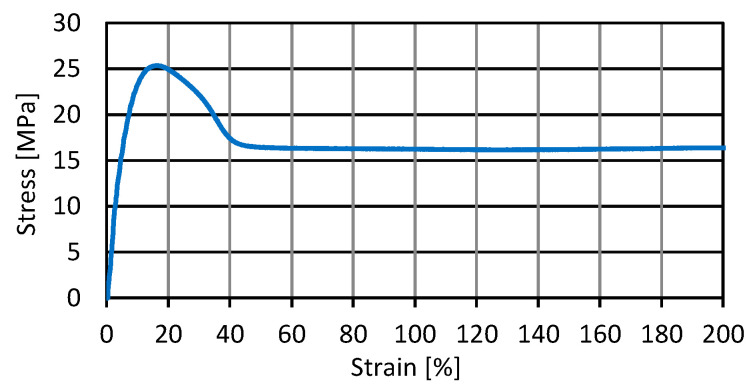
Diagram of the relationship between tensile strength and elongation of a high-density polyethylene pipe (HD-PE).

The tensile strength of the pipe material carried out according to the standard PN-EN ISO 527-2: 2012 [30] was determined (Figure 4). The samples were tested using an electromechanical tensile testing machine type ZWICK100 (ZwickRoell, August- Nagel- Straße 11, 89079 Ulm, Germany), with a measuring range of 0–100 kN.

### 2.2. Experimental Research of the Influence of External Load on the Deformation of Polyethylene Pipe

**Figure 5:**
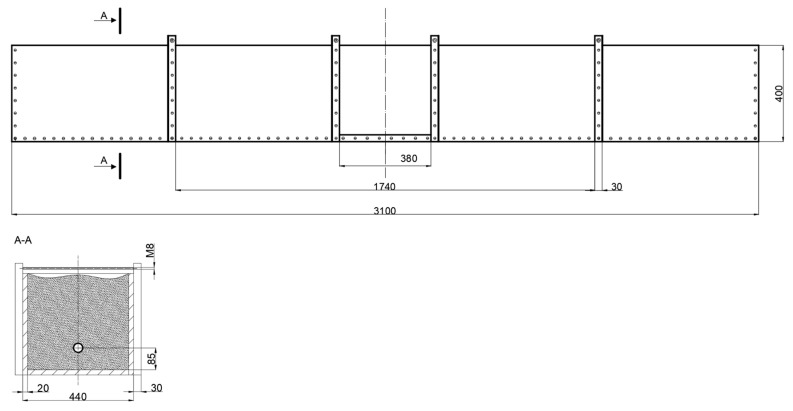
Schematic of the test stand. (Units in mm).

The experimental research was carried out on a test stand made of oriented strand board (the approximate dimensions of the box were 3100 mm × 400 mm). A plastic peephole was installed in the central part of the test stand’s side wall. The stand was used to perform an experiment of simulating the actual conditions that prevail during pipe deformation caused by the load. The test pipe was placed in the box on a sand bed. The same type of sand was used for backfilling of the pipe. The loads of 500, 1000, 1500, 1500, and 2000 N were applied to the upper surface of the backfill in the central part of the box. The value of deformation was recorded at the moment of pipe load. A scheme of the box is shown in Figure 5.

Experimental investigations were carried out using a high-density polyethylene (HD-PE) pipe (pipe dimensions: length of 2300 mm, external diameter of 40 mm, and pipe wall thickness of 3.7 mm).

On the lower and upper surface of the pipe, the strain gauges were placed at equal intervals of 120 mm with numbers from 1 to 20 (the sensor marked as 10 was located in the central part of the pipe). In this research, electrical and hose strain gauges by Microtechna (manufacturer of the strain gauges) were used, which were glued to the pipe walls. The sensors were used to record changes in the lateral strains of the pipe at different external loads. The strain gauges were made of one piece of wire which was glued to the paper or foil in a hose manner.

In the resistance strain gauges used in the experiment, the strains were measured based on the relationship between electrical resistance and the length of the wire—Equation (2):

(2) $$R=\delta \frac{L_{d}}{A}$$

where:

R—the electrical resistance of the wire, Ω;
  δ—specific resistance of the wire, Ω;
  L_d_—wire length, mm;
  A—wire cross-sectional area, mm.

The relative increment of strain gauge resistance is described in Equation (3):

(3) $$\frac{\Delta R}{R_{1}}=\frac{\Delta \delta }{\delta }+\frac{\Delta L_{d}}{L_{d}}-\frac{\Delta A}{A}$$

where:

$\frac{\Delta \delta }{\delta }$—relative increment of specific resistance;
  $\frac{\Delta L_{d}}{L_{d}}$—relative strain of the wire;
  $\frac{\Delta A}{A}$—relative change in the cross-section of the wire.

In order to determine ∆A/A, a square with ABCD sides was determined on the cross-section of the wire. At the load applied, the lengths of the square sides with values of (1 + ε_x_) and (1 + ε_y_) were deformed. The square cross-sectional area was initially equal to A_k_ = 1, and after deformation, it was as described in Equation (4):

A’_k_ = (1 + ε_x_) − (1 + ε_y_) (4)

where:

ε_x_—strain of the cross-section in the x-direction;
  ε_y_—strain of the cross-section in the y-direction.

The relative change in the wire cross-section is described in Equation (5):

(5) $$\frac{\Delta A}{A}=\frac{{A^{\prime }}_{k}-A_{k}}{A_{k}}=\frac{\left(1+\varepsilon_{x}\right)\left(1+\varepsilon_{y}\right)-1}{1}=\varepsilon_{x}+\varepsilon_{y}+\varepsilon_{x}\varepsilon_{y}$$

Excluding the product $\varepsilon_{x}\varepsilon_{y}$ as an infinitesimally small quantity, and taking into account the fact that during stretching, the strain gauge wire is in a unidirectional state of stress, that is, ε_x_ = ε_y_ = −ν_ε_, the relative change in the wire cross-section can be obtained using Equation (6):

(6) $$\frac{\Delta A}{A}=-2\nu \varepsilon$$

The relative increase in strain gauge resistance can be written as Equation (7):

(7) $$\frac{\Delta R}{R}=\left(\frac{\left(\frac{\Delta \delta }{\delta }\right)}{\varepsilon }+1+2\nu \right)\varepsilon$$

The relative strain is described in Equation (8):

(8) $$\varepsilon =\frac{1}{\left(1+2\nu +\frac{\Delta \delta /\delta }{\varepsilon }\right)}\frac{\Delta R}{R}$$

The value of the denominator in Equation (8) is the constant k, called the strain gauge constant, according to Equation (9) [31]:

(9) $$k=1+2\nu +\frac{\Delta \delta /\delta }{\varepsilon }$$

The relative strain can be written as Equation (10):

(10) $$\varepsilon =\frac{1}{k}\left(\frac{\Delta R}{R}\right)$$

where:

k—strain gauge constant: 2.15;
  ΔR—relative increase in electrical resistance;
  R—the wire’s electrical resistance.

The value of the constant k in in Equation (9) depends on the sensor wire material, and it ranges between 1.6 and 3.6. For strain gauges used in this experiment, the constant k was 2.15 (value of k is provided by the manufacturer of the strain gauges).

## 3. Results and Discussion

Below are the results of the numerical simulation and experimental tests of polyethylene pipe loading.

### 3.1. Numerical Analysis of the Influence of External Load on the Deformation of Polyethylene Pipe

**Figure 6:**
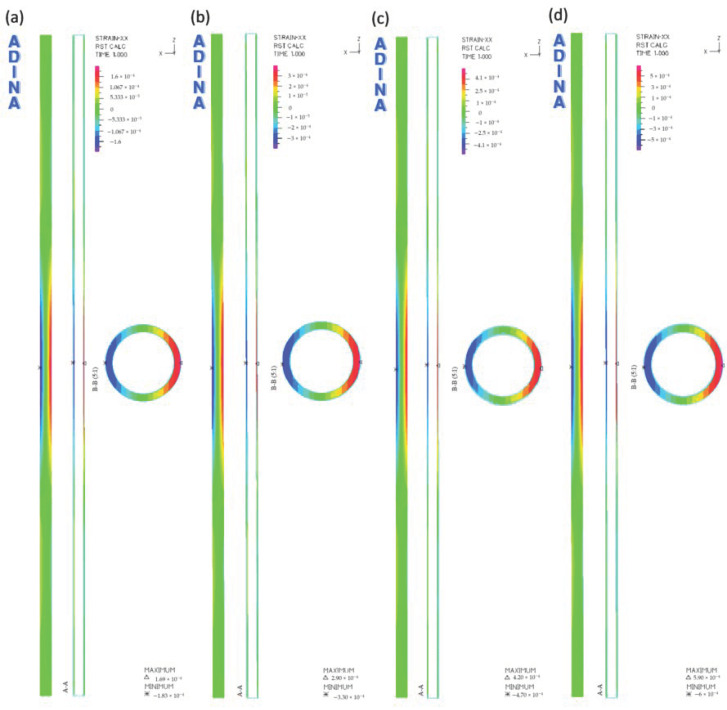
The pipe longitudinal deformations ε_x_ at the following loads: (**a**) 500 N, (**b**) 1000 N, (**c**) 1500 N, (**d**) 2000 N.

The results of the numerical analysis, illustrating the distribution of longitudinal strains of the tested pipe for different values of external load, are presented in Figure 6. In order to accurately illustrate the distribution of deformations, two sections were made on the models: transverse in the central part of the pipe and longitudinal along the pipe’s entire length.

The nature of the longitudinal deformations of the pipe is the same for each load case. Considering the top surface of the pipe, the following deformations were noted: negative in the middle part and positive at the ends of the pipe model. On the pipe’s lower surface, the following deformations were noted: positive in the middle part and negative at the ends of the pipe model.

**Figure 7:**
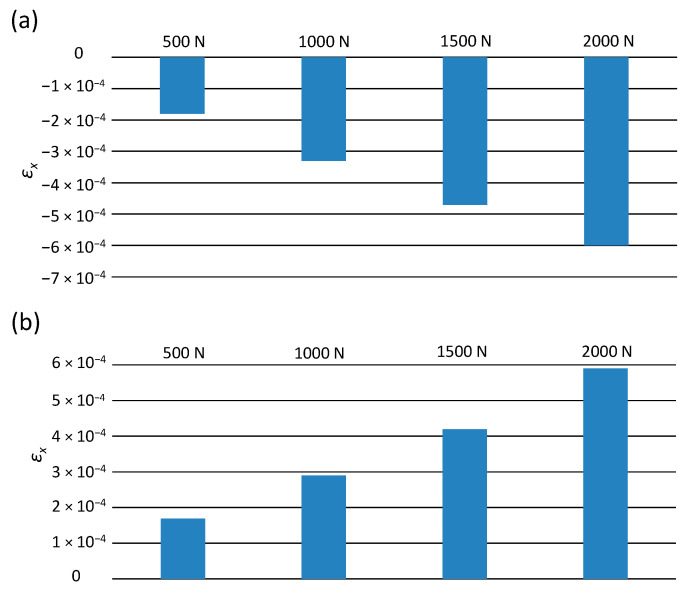
The maximum values of longitudinal deformations ε_x_: (**a**) on the top surface of the pipes, (**b**) on the bottom surface of the pipes.

Figure 7 shows the values of the largest determined deformations of the tested pipes, depending on the external load.

Figure 7 shows that an increase in deformations is directly proportional to the external load increasing. For example, at the load of 500 N, the following values of maximum deformation occur: −1.8 × 10^−4^ on the upper surface, and 1.7 × 10^−4^ on the pipe’s lower part. At the load of 2000 N, this value is approximately 3.3 times greater.

**Figure 8:**
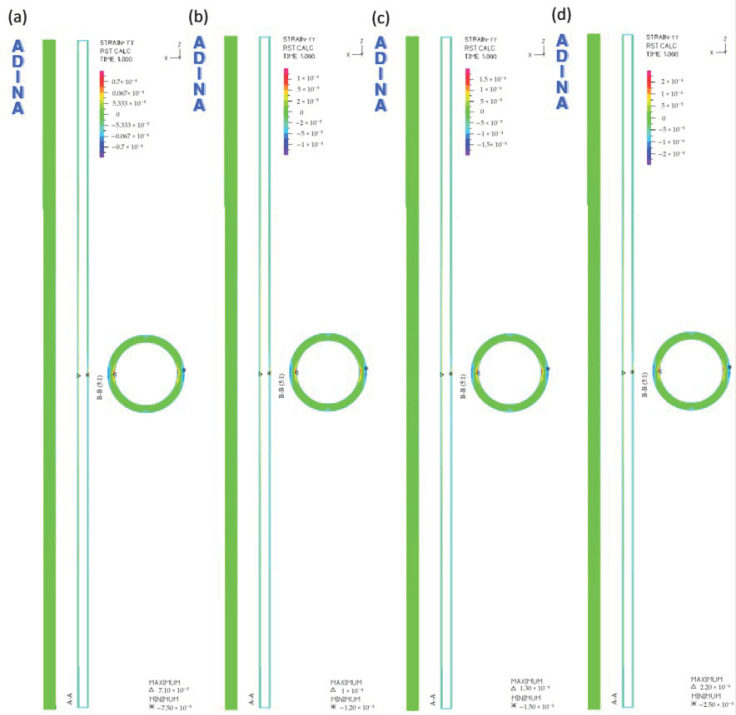
The pipe transverse deformations ε_y_ at the following loads: (**a**) 500 N, (**b**) 1000 N, (**c**) 1500 N, (**d**) 2000 N.

Figure 8 presents the results obtained from the numerical analysis, illustrating the distribution of the longitudinal strains of the pipe at the considered values of the external load. The model also includes the cross-section in the central part of the pipe and the longitudinal section along its entire length.

The distribution of transverse deformations of the tested pipe is of the same nature in each analyzed load case. On the lower and upper surface of the pipe, in its middle part, the deformation values are negative, while at the pipe ends, the values are positive.

**Figure 9:**
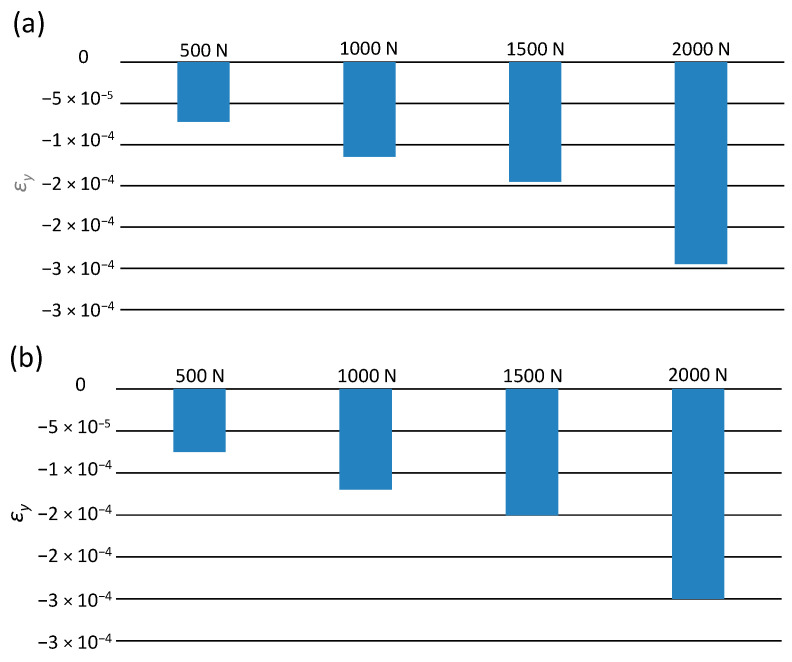
The maximum values of transverse deformations ε_y_: (**a**) on the upper surface of the pipes, (**b**) on the lower surface of the pipes.

Figure 9 shows the values of the maximum deformations of the tested pipe, depending on the external load.

The highest values of negative deformations in each load variant occur in the central part of the pipe. Along with the increase in the value of the soil load, the lateral deformation increased. It can be concluded that, as in the case of longitudinal deformations, the smallest values of pipes transverse deformations occur at the load of 500 N. On the upper surface of the pipe, the value is equal to −7.3 × 10^−5^, while in the lower part, it is equal to −7.5 × 10^−5^. In the case of loading of 2000 N, an increase in the deformation value of −2.8 × 10^−4^ at the top was observed, while an increase in the deformation value of −3 × 10^−4^ at the bottom of the pipe in relation to the load of 500 N was noted. The values of longitudinal and transverse deformations are small, falling within the yield point of polyethylene [32,33]. Therefore, in the operating conditions of the pipelines, such soil loading would not cause the pipe to break or disturb the transport of the medium.

### 3.2. Comparative Analysis of Numerical Simulation and Experimental Research of the Influence of the External Load on Polyethylene Pipe Deformation

**Figure 10:**
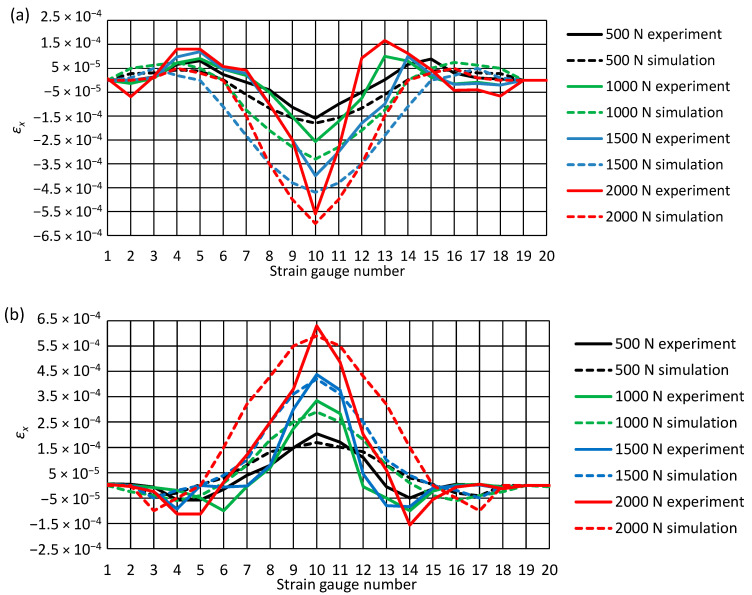
Longitudinal deformations of the pipe ε_x_ at different external loads: (**a**) top of the pipe, (**b**) bottom of the pipe.

Figure 10 present the results of experimental tests of the pipe longitudinal strain measurements, using electric resistance strain gauges as shown. The horizontal axis of the charts shows the numbers of successive strain gauges attached to the pipe’s surface, while the ordinate axis shows the values of the recorded strains. For comparison, the graphs also show the longitudinal deformations of the pipe obtained during the numerical analysis.

The results of experimental tests of longitudinal deformation of the pipe at the considered loads confirmed the results obtained during the numerical analysis, and they have the same trend. The highest negative deformation appeared to occur on the pipe’s upper surface, while the maximum positive deformation was recorded in its lower part.

**Figure 11:**
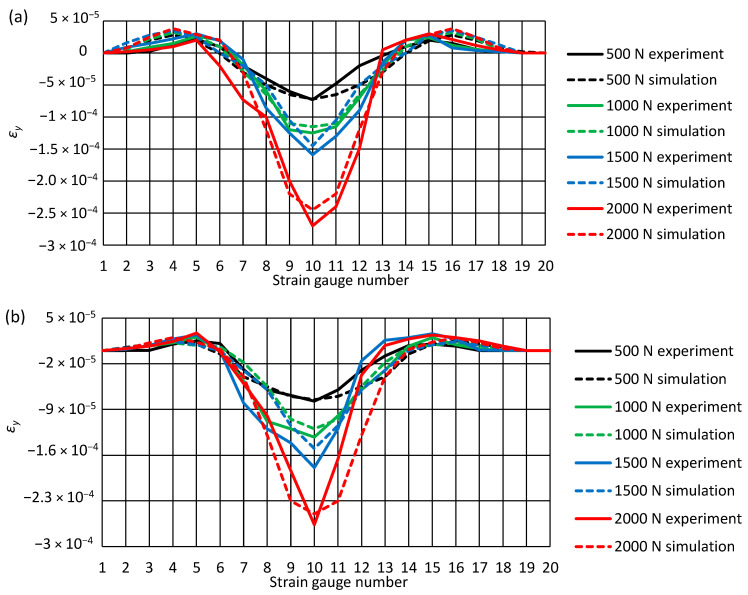
Transverse deformations of the pipe ε_y_ at different external loads: (**a**) top of the pipe, (**b**) bottom of the pipe.

Figure 11 shows the results of the measurement of the transverse deformations of the pipe obtained during the experimental tests. As in the case of the pipe longitudinal deformation analysis, the numbers of successive strain gauges are marked on the abscissa axis of the graphs, while the values of the registered deformations of the pipe are located on the ordinate axis. The diagrams also show the results obtained during the numerical analysis.

As in the case of longitudinal deformations of the pipe, the results of the experimental tests also agree with the results obtained from the computer simulation. The nature of deformations is the same in each analyzed case. On the lower and upper surface of the pipe, the greatest negative values of deformation always occur in the center of the pipe. In turn, deformations with a positive sign are located at the pipe ends. In each load variant, the positive deformations are slightly greater on the pipe’s upper surface.

Table 1 summarizes the differences in the deformation values of ε_x_ and ε_y_ observed in the results obtained from the simulation and the experiment for the central part of the pipe (where the pipe’s highest deformation values occur).

The largest recorded difference in deformation ε_x_ between the results obtained from the computer simulation and the results of experimental tests concerns the upper surface of the pipe for a load of 1000 N. The maximum value of deformation obtained from the experiment is 7.43 × 10^−5^ smaller than the value obtained from the simulation. The greatest difference in the value of the maximum deformation ε_y_ was recorded for the lower surface of the pipe at the load of 1500 N. The difference in values between the experimental test and the numerical analysis is 2.98 × 10^−5^.

The slight differences in the obtained results confirm the high accuracy of the computer simulation and the experimental research.

## 4. Conclusions

It is necessary to conduct research on changes in pipe properties as a result of degradation processes. The results of such studies may contribute to the prediction of failure-free operation of pipelines, as well as earlier planning of their repairs or replacements, which is reflected in the reduction of downtime in the supply or receipt of utilities, both from households and production companies.

Conducting experimental tests and numerical simulations allowed for a comparative analysis between them and recognition of the impact of the proposed values of polyethylene pipes’ load on the change in their deformation. The high consistency of the results of computer simulations with the results of experimental tests obtained in the work indicates the appropriate application of the models in the problem under consideration (soil modeling—elastic–ideally plastic Coulomb–Mohr model; pipe modeling—elastic–isotropic model).

The authors plan to conduct further research on the change in the mechanical properties of polyethylene pipes after aging, corresponding to operation of a few years (2, 5 and 10 years).

## Figures and Tables

**Table 1:** Comparison of the differences in the values of longitudinal deformations ε_x_ and transverse deformations ε_y_ observed in the results of computer simulation and experimental tests.

	500 N	1000 N	1500 N	2000 N
		ε_x_		
top of the pipe	2.11 × 10^−5^	7.43 × 10^−5^	7.26 × 10^−5^	4.34 × 10^−5^
down the pipe	3.43 × 10^−5^	4.23 × 10^−5^	1.78 × 10^−5^	4.31 × 10^−5^
		ε_y_		
top of the pipe	1.13 × 10^−5^	1.06 × 10^−5^	1.43 × 10^−5^	2.56 × 10^−5^
down the pipe	2.35 × 10^−5^	1.28 × 10^−5^	2.98 × 10^−5^	1.66 × 10^−5^

## Data Availability

Not applicable.
